# Radioresistant human lung adenocarcinoma cells that survived multiple fractions of ionizing radiation are sensitive to HSP90 inhibition

**DOI:** 10.18632/oncotarget.6248

**Published:** 2015-10-27

**Authors:** Roberto Gomez-Casal, Michael W. Epperly, Hong Wang, David A. Proia, Joel S. Greenberger, Vera Levina

**Affiliations:** ^1^ University of Pittsburgh Cancer Institute, Pittsburgh, PA, USA; ^2^ Department of Medicine, University of Pittsburgh, Pittsburgh, PA, USA; ^3^ Department of Radiation Oncology, University of Pittsburgh, Pittsburgh, PA, USA; ^4^ Department of Biostatistics, University of Pittsburgh, Pittsburgh, PA, USA; ^5^ Synta Pharmaceuticals Corp., Lexington, MA, USA; ^6^ Current address: Hillman Cancer Center, University of Pittsburgh Cancer Institute, Pittsburgh, PA, USA

**Keywords:** lung adenocarcinoma, fractionated ionizing radiation, radioresistance mechanisms, DNA repair, heat shock protein-90

## Abstract

Despite the common usage of radiotherapy for the treatment of NSCLC, outcomes for these cancers when treated with ionizing radiation (IR) are still unsatisfactory. A better understanding of the mechanisms underlying resistance to IR is needed to design approaches to eliminate the radioresistant cells and prevent tumor recurrence and metastases. Using multiple fractions of IR we generated radioresistant cells from T2821 and T2851 human lung adenocarcinoma cells. The radioresistant phenotypes present in T2821/R and T2851/R cells include multiple changes in DNA repair genes and proteins expression, upregulation of EMT markers, alterations of cell cycle distribution, upregulation of PI3K/AKT signaling and elevated production of growth factors, cytokines, important for lung cancer progression, such as IL-6, PDGFB and SDF-1 (CXCL12). In addition to being radioresistant these cells were also found to be resistant to cisplatin.

HSP90 is a molecular chaperone involved in stabilization and function of multiple client proteins implicated in NSCLC cell survival and radioresistance. We examined the effect of ganetespib, a novel HSP90 inhibitor, on T2821/R and T2851/R cell survival, migration and radioresistance. Our data indicates that ganetespib has cytotoxic activity against parental T2821 and T2851 cells and radioresistant T2821/R and T2851/R lung tumor cells. Ganetespib does not affect proliferation of normal human lung fibroblasts. Combining IR with ganetespib completely abrogates clonogenic survival of radioresistant cells.

Our data show that HSP90 inhibition can potentiate the effect of radiotherapy and eliminate radioresistant and cisplatin -resistant residual cells, thus it may aid in reducing NSCLC tumor recurrence after fractionated radiotherapy.

## INTRODUCTION

Lung cancer is the leading cause of cancer death in the United States, with non-small cell lung cancer (NSCLC) constituting more than half of all lung cancer cases [1]. Adenocarcinoma (AC) is the major histological subtype of non-small cell lung cancer. Radiotherapy (RT), either alone or in combination with surgery or chemotherapy is among the primary treatment protocols for NSCLC [2–4]. Unfortunately, the therapeutic outcomes in many cases are not satisfactory. Tumor radioresistance leads to a reduction in the efficiency of RT with corresponding tumor recurrence and metastasis [2–4]. Therefore, it is important to investigate the cellular mechanisms leading to this loss of radiosensitivity and to discover potential therapeutic compounds which might significantly improve the efficacy of these treatments and prevent metastasis.

A large number of studies have demonstrated that radiosensitivity of cells and tissues depends on many factors such as double strand break (DSB) repair via homologous recombination (HR) and nonhomologous end-joining (NHEJ), proliferation rates, radiation-induced modification of the genes involved in cell cycle progression, and apoptosis [5–7]. Phosphorylated histone H2AX at Ser^139^ (γH2AX) foci has been established as a sensitive indicator of DNA DSBs [8]. p53-binding protein 1 (53BP1) has emerged as a central component of the chromatin-based DSB signal, acting as a mediator and an effector of the DSB response [9]. The numbers of γH2AX and 53BP1 foci are believed to parallel the numbers of DSBs found in the cells [8–11]. Experimental studies on cultured NSCLC cells have revealed that radioresistance in lung tumor cells might be also associated with activation of other mechanisms, such as PI3K/Akt signaling [12, 13], and JAK2/STAT3/Bcl2/Bcl-XL survival pathways [14]. These findings suggest that the mechanisms of radioresistance are not regulated by a single gene but involve complex multi-gene interactions [6].

HSP90 is a chaperone protein important in the stabilization and trafficking of proteins (clients) involved in cancer progression and radiation and drug resistance [15]. HSP90 is found to be over expressed in a variety of cancers, including lung cancer [16]. Inhibition of HSP90 results in client protein destabilization and degradation via the ubiquitin-proteasome pathway. Notable client proteins include hypoxia-inducible factor-1 (HIF-1) [17], signal transducer and activator of transcription (STAT-3) [18], PI3K-AKT-mTOR, DNA damage repair and cell cycle regulation pathways [19, 20] which are essential for cancer growth. HSP90 inhibitor 17-AAG blocked radiation-induced stabilization of HIF-1a and increased radiosensitivity of lung cancer cells [21]. HSP90 inhibitors are currently being developed as anticancer agents and have shown encouraging signs of activity in molecularly defined subgroups of solid tumors [22, 23].

Ganetespib is a unique resorcinol inhibitor of HSP90 that is currently in clinical trials for a number of human cancers [24]. Ganetespib has shown preclinical activity against NSCLC models, including those driven by mutant EGFR, rearranged ALK, and/or mutant KRAS [4, 25, 26], and has been shown to potentiate the effect of taxanes and PI3K/mTOR inhibitor BEZ235 in NSCLC models [25, 26]. Ganetespib has a manageable side effect profile, and has demonstrated promising activity in heavily pretreated patients with advanced NSCLC [27]. We recently reported that ganetespib treatments had a radiosensitizing effect in human lung adenocarcinoma cells both *in vitro* and *in vivo* studies [28]. In these studies, we sought to determine if ganetespib would be able to overcome radio-and cisplatin-resistance which has developed in NSCLC cells that survived multiple fractions of IR and radiosensitize or eliminate radioresistant residual cells. These proofs of concept studies show that HSP90 inhibition offers a potential strategy for enhancing the effect of radiotherapy and reducing radioresistance.

## RESULTS

### Establishment and characterization of T2821/R and T2851/R radioresistant cells

T2821 and T2851 human lung adenocarcinoma cell lines established from surgical samples [28] were used to generate IR-resistant cell lines. T2851 cells harbor an EGFR mutation (exon 21, L858R mutation), whereas T2821 cells have no major known oncogenic mutations but are a known lung AC cell line (wt EGFR, wt BRAF, wt KRAS, no ALK fusion).

**Figure 1:**
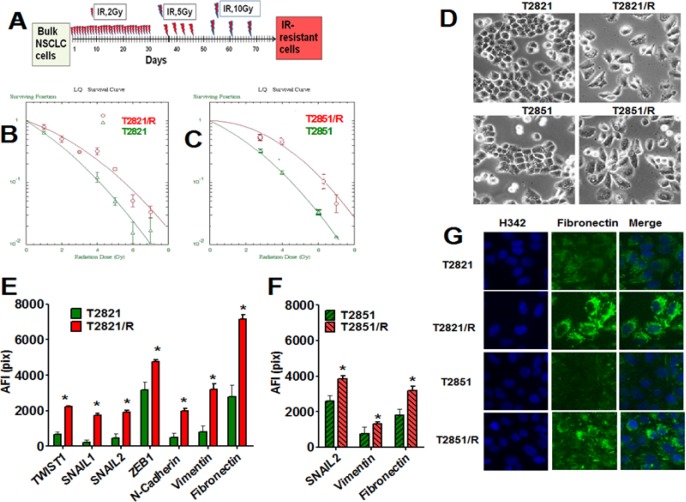
Generation of IR-resistant lung adenocarcinoma cells surviving multiple fractions of IR (**A**) Strategy for the generation of T2821/R and T2851/R radio resistant residual lung adenocarcinoma cells. (**B**) (**C**) T2821/R and T2851/R cells show higher clonogenic survival after IR-treatment. Cells were suspended, irradiated (0–10 Gy) and plated. On the seventh day after IR treatment, cells were fixed and clonogenic survival was estimated. Radiation survival curves show IR-sensitivity of T2821 and T2821/R (B), T2851 and T2851/R (C) cells. (**D**) Morphology changes in T2821/R and T2851/R cells. Phase contrast images of T2821 and T2821/R cells, as well as T2851 and T2851/R cells are shown. E-G. Analysis of EMT associated proteins expression in radioresistant and parental cells. Cells were grown in 96 well plates, fixed and stained for TWIST1, SNAIL1, SNAIL2, ZEB1, N-cadherin, Vimentin and Fibronectin and with Hoechst 33342. Cell images were analyzed using HCA/HCS methods. The total average fluorescence intensities of proteins determined in T2821 and T2821/R cells (**E**) and T2851 and T2851/R cells (**F**) are shown. Only proteins with significant differences between parental and IR-resistant cells are shown. (**G**) Images of T2821, T2821/R, and T2851/R cells stained for fibronectin (green) and with Hoechst 33342 (blue) are shown. *denotes significant differences between groups of tumor tissues at *p* < 0.05.

When the cells reached about 60% confluence IR treatments were initiated. We applied multiple increasing intensity fractions of IR. T2821 and T2851 cells were irradiated 20 times (once a day) with the dose of 2 Gy, then 4 times with the dose of 5 Gy and 3 times with the dose of 10 Gy (Figure 1A). When cells reached 90% of confluence, they were subcultured. Untreated parental T2821 and T2851 cells were cultured under the same conditions without irradiation. Cells were cultured in adherent conditions in complete cell culture media supplemented with FBS. Cells which survived multiple fractions of IR treatment (in total, 90 Gy) were named as T2821/R and T2851/R, respectively. T2821, T2851, T2821/R and T2851/R cells were collected, and stocks of the frozen cells were prepared for further study.

First, we determined plating efficiency of parental T2821, T2851 cells and T2821/R and T2851/R cells growing in physiologically normal conditions without irradiation. T2821/R and T2851/R cells showed lower plating efficiency compared to respective parental cells (Table 1). The “classical” clonogenic survival assay was employed to compare radiosensitivity of T2821/R and T2851/R cells with T2821 and T2851 parental cells. T2821/R and T2851/R cells demonstrated significantly higher levels of the clonal survival after irradiation in comparison with the parental T2821 and T2851 cells (Figure 1B, 1C, and Table 1).

**Table 1:** Characterization of lung adenocarcinoma cells survived multiple fractions of IR

		T2821	T2821/R	T2851	T2851/R
1.	Plating efficiency	66.98 ± 1.949	*50.82 ± 1.57	58.07 ± 3.99	*53.76 ± 2.576
2.	IR sensitivity, D_0_	1.279 ± 0.12	*1.546 ± 0.060	1.351 ± 0.07	*1.659 ± 0.039
3.	Cisplatin sensitivity, IC_50_	3.67 ± 0.31	*8.31 ± 0.66	7.29 ± 0.69	*9.26 ± 0.92

1. Plating efficiency of the tumor cells is presented as a % of the initial cell numbers ± standard deviation (SD).

2. Radiation sensitivity of lung adenocarcinoma cells. Results of clonogenic survival assay were calculated with linear quadratic and single hit multi-target models and presented as D_0_ – Gy, (the dose required to reduce the fraction of survived cells to 37%) ± SD.

3. Sensitivity to cisplatin of lung adenocarcinoma cells. Results of MTT assay were calculated and presented as IC50 – μM, (the concentration of cisplatin required to reduce the fraction of survived cells to 50%) ± SD.

*P* < 0.05 (compared to respective parental cell group).

Next we tested the effect of cisplatin on the viability of T2821, T2851 and T2821/R and T2851/R cells using MTT assay. Both T2821/R and T2851/R radioresistant cell lines also showed significant resistance to cisplatin as compared to the parental T2821 and T2851 cells (Table 1).

Radioresistant and parental tumor cells display differing cellular morphologies. While parental T2821 and T2851 cells showed tight cell- cell junctions as expected for epithelial cells, while the T2821/R and T2851/R cells exhibited a more spindle–like morphology, and showed a loss of cell-to cell junctions with an increase in cellular scattering (Figure 1D). To determine whether the spindle shape of resistant cells is associated with the epithelial- to mesenchymal transition (EMT) phenotype, we assessed the expression of the major transcription factors involved in regulation of EMT in lung AC cells; SNAIL1, SNAIL2 (SLUG), TWIST1, and ZEB1 [29–31]. In addition we looked at EMT associated proteins such as N-cadherin, vimentin and fibronectin [29, 30, 32, 33]. We found that multiple EMT associated proteins, such as SNAIL1, SNAIL2, TWIST1, ZEB1, N-cadherin, vimentin and fibronectin, are significantly upregulated in T2821/R cells as compared to parental T2821 cells (Figure 1E and 1G).

SNAIL2, vimentin and fibronectin were significantly upregulated in T2851/R cells compared to parental T2851 cells, while other proteins were expressed similarly as compared to parental T2851 cells (Figure 1F and 1G). These data demonstrate that both IR treated adenocarcinoma cell lines display upregulation of EMT associated proteins; however changes in the pattern of the gene expression is different in the two resistant cell lines.

**Figure 2:**
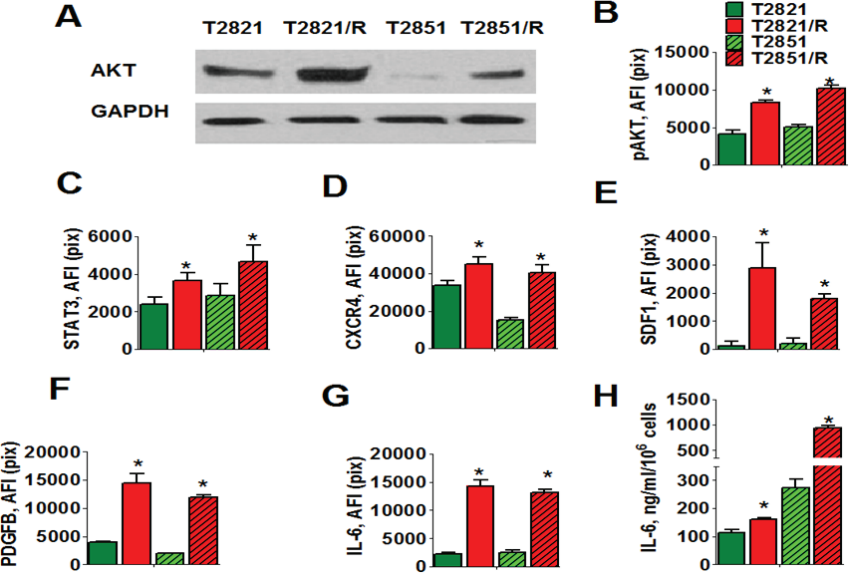
Cell signaling changes associated with IR-resistance (**A–D**). Radioresistant lung adenocarcinoma cells express increased levels of AKT, p-AKT, STAT3, and CXCR4 receptors. (**A**) Western blots analysis of AKT expression in radioresistant T2821/R and T2851/R cells and parental T2821 and T2851 cells. Adenocarcinoma cells were grown in physiologically normal conditions; cell lysates were prepared and immunoblotted using antibodies against AKT. GAPDH was included as a control. (**B–D**). T2821, T2851, T2821/R, and T2851/R cells were grown in 96 well plates, fixed and stained for phospho-AKT, STAT3 and CXCR4 (SDF-1 receptor), and with Hoechst 33342. Cell images were acquired using the Cellomics ArrayScan HCS Reader (40X objective) and analyzed using the Compartment Analysis BioApplication Software Module. The total average fluorescence intensities of p-AKT (**B**), STAT3 (**C**) and CXCR4 (**D**) in the parental cells (green), and IR-resistant cells (red) are shown. Each point represents average intensities (pixels) estimated for 3000 cells. (**E–H**) Radioresistant cells are producing elevated levels of IL-6, PDGFB and SDF-1 growth factors. (**E–G**). Cells were grown in 96-well plates in cell culture media supplemented with monensim, 2 μM, for 48 hours. Cells were fixed, permeabilized and immunofluorescently stained for PDGFB, IL-6 and SDF-1 (CXCL12) and with Hoechst 33342. Cell images were acquired using the Cellomics ArrayScan HCS Reader (20X objective) and analyzed using the Compartment Analysis BioApplication Software Module. The total average fluorescence intensities of SDF-1 (E), PDGFB (F), and IL-6 (G) in the parental cells (green) and IR-resistant cells (red) are shown. The fluorescence intensities of respective IgG controls were subtracted. Each point presents average intensities (pixels) estimated for 3000 cells. H. Cells were cultivated in 96 well plates for 72 h in complete RPMI 1640 medium; samples of conditioned media were collected. Cells were fixed, stained with Hoechst 33342, and cell numbers were determined using image cytometry. Concentration of IL-6 was analyzed using ELISA. Data are presented as pg/10^6^ cells/ml. *denotes significant differences between groups of tumor tissues at *p* < 0.05

AKT/protein kinase B is involved in radiation resistance in NSCLC [12, 34]. We compared expression of AKT in parental T2821 and T2851 cells and radioresistant T2821/R and T2851/R cells cultured under physiologically normal conditions. T2821/R and T2851/R cells express higher levels of total AKT (Figure 2A), as well as activated p-AKT (Figure 2B). than parental T2821 and T2851 cells. Upregulation of the IL-6/STAT3 pathway is associated with lung cancer progression [35]. EMT in melanoma and pancreatic cancers is associated with the platelet-derived growth factor B (PDGFBB) upregulation [36, 37]. The inflammatory cytokine interleukin-6 (IL-6) and stromal derived factor1 (SDF-1/CXCL12), are believed to play key roles in tolerance and acquired resistance to the conventional therapies. These growth factors might support survival and proliferation of tumor cells through autocrine and paracrine mechanisms [38–42].

Therefore, we analyzed expression of STAT3 and production of the ligands IL-6, PDGFBB, and SDF-1 along with the level of expression of their receptors (PDGFR-beta, IL-6R, GRP130 and CXCR4) in parental and radioresistant lung tumor cells (Figure 2C–2H). Monensim, 2 μM, was used to block in-situ cytokine secretion in this assay [43]. Intracellular immunofluorescent staining revealed that T2821/R and T2851/R cells express higher levels of STAT3 (Figure 2C) than parental T2821 and T2851 cells. While PDGFR-beta, IL-6R, GRP130 receptors were expressed in equal levels in parental and radioresistant cells (data not shown). CXCR4 receptors were found to be significantly upregulated in T2821/R and T2851/R cells in comparison with parental T2821 and T2851 cells (Figure 2D). T2821/R and T2851/R cells produce dramatically higher quantities of SDF-1, PDGFB and IL-6 proteins than parental T2821 and T2851 cells (Figure 2E–2G). To further verify that IR-resistant T2821/R and T2851/R cells produce higher level of IL-6 than the parental cells (T2821 and T2851) we analyzed the concentration of IL-6 found in samples of culture media conditioned by these cells using ELISA. As shown in Figure 2H, all four cell lines produce very high level of IL-6, with concentration of IL-6 in culture media samples varying from 100 ng/ml/10^6^ cells to more than 1000 ng/ml/10^6^ cells. While both parental and resistant cells produce IL-6, T2821/R and T2851/R cells were both found to produce a higher amount of IL-6 as compared with their parental cells. In general, this finding is consistent with our observations using HCS and HCA methods for detection of intracellular cytokines (Figure 2H). The overall lower level of IL-6 detected in the T2821 derived cells versus the T2851 derived cells is probably associated with different abilities of the cell lines to secret cytokines.

### Analysis of cellular growth rates and cell doubling times in radioresistant and parental AC cells growing under physiologically normal condition

Cellular growth rates and estimated doubling times in parental T2821 and T2851 cell lines and radioresistant T2821/R and T2851/R cells were compared. The mean and standard deviation for number of cells per well and estimation and comparison of cell doubling times are summarized in the Table 2. T2821, T2851 and T2821/R cells were found to have a similar relatively high growth rates (Table 2). In contrast, T2851/R cells had a significantly longer doubling time than T2821 and T2851 cells (*p* = 0.0184 and 0.0282 respectively).

**Table 2:** Comparison of cell doubling times in parental and radioresistant lung tumor cells

Cell Group	Time, h					Cell's doubling time (h)	*p*-value for the comparison of doubling times with the *z*-test
	0	8	24	48	72		
**T2821**	0.26 (0.07)	0.48 (0.22)	1.70 (0.29)	5.88 (0.45)	11.55 (1.84)	12.80 (0.79)	
**T2821/R**	0.27 (0.11)	0.51 (0.18)	1.56 (0.17)	5.18 (0.42)	9.05 (0.40)	13.77 (0.92)	*P* = 0.4237 compared to T2821
**T2851**	0.31 (0.10)	0.49 (0.28)	2.20 (0.41)	6.22 (0.55)	12.10 (0.29)	13.00 (0.95)	*P* = 0.8704 compared to T2821 *P* = 0.5623 compared to T2821/R
**T2851/R**	0.28 (0.06)	0.88 (0.44)	2.13 (0.19)	4.51 (0.36)	5.45 (0.40)	17.84 (1.99)	*P* = 0.0184 compared to T2821 *P* = 0.0635 compared to T2821/R *P* = 0.0282 compared to T2851

The mean and standard deviation (in parentheses) for number of cells per well at each time of measurement are in the first several column. Cell doubling times and their standard errors, as well as the comparison of cell doubling times, are in the last two columns.

### Effect of gamma-radiation on cell cycle progression in radioresistant and parental lung AC cells

**Figure 3:**
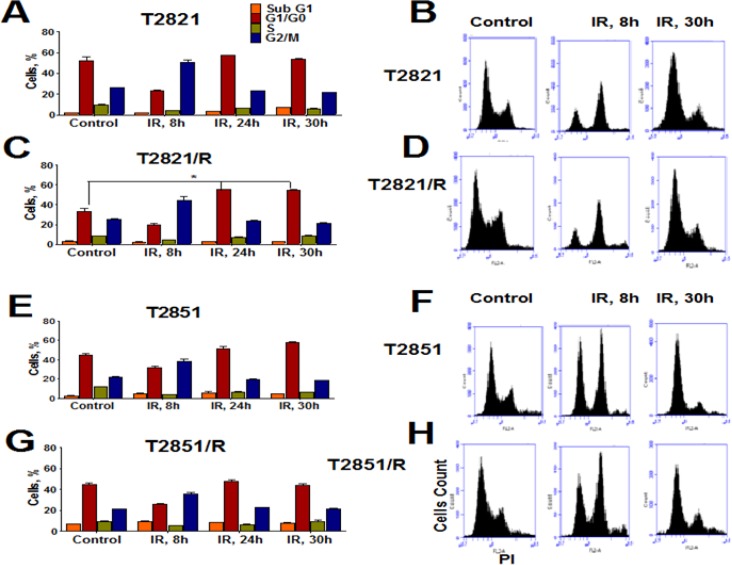
Effect of gamma-radiation on cell cycle distribution in radioresistant and parental lung adenocarcinoma cells Exponentially growing parental T2821 and T2851 cells and radioresistant T2821/R, and T2851/R cells were irradiated (0, or 5 Gy) and fixed after 0, 8, 24 and 30 hours post-radiation. Cells were stained with PI and the cell cycle distribution was measured by flow cytometry. Cell cycle distributions for control cells were similar at 0, 8, 24, and 30 hours, therefore only control cells at 0 point are presented. Quantification of cells in phases of cell cycle are shown for T2821 (**A**) T2821/R (**C**), T2851 (**E**) and T2851/R (**G**) cells. Data are presented as the mean ± SD of three experiments. Representative flow cytometry histograms for T2821 (**B**) T2821/R (**D**) T2851 (**F**) and T285/R (**H**) cells determined for control cells or irradiated cells at 8 hours and 30 hours post-radiation are shown. *denotes significant differences between groups of tumor tissues at *p* < 0.05

To compare potential cell cycle effects, parental T2821 and T2851 cells and the radioresistant T2821/R and T2851/R cells were gamma-irradiated (0, or 5 Gy) and grown for 0, 8, 24 and 30 h after IR treatment (Figure 3). The cell cycle distributions in control (0 Gy) cells were found to be similar at all time points (0, 8, 24 and 30 h), therefore in Figure 3 we present only cell cycle data for AC cells at time point 0. Cell cycle analysis revealed G2 accumulation in IR-treated cells, which was associated with a concomitant decreased of S phase and G1 fraction at 8 h post-radiation, and G2 fractions were decreased at 24 and 30 h post-radiation in each of the parental and radioresistant cell lines.

These data suggest that radioresistance in the cells surviving multiple fractions of IR treatment results from differential responses to the IR treatments. While the slow growing T2851/R cells showed only G2/M arrest after IR treatment.

### Profiling of DNA repair genes expression in parental and radioresistant cells growing under physiologically normal condition

**Figure 4:**
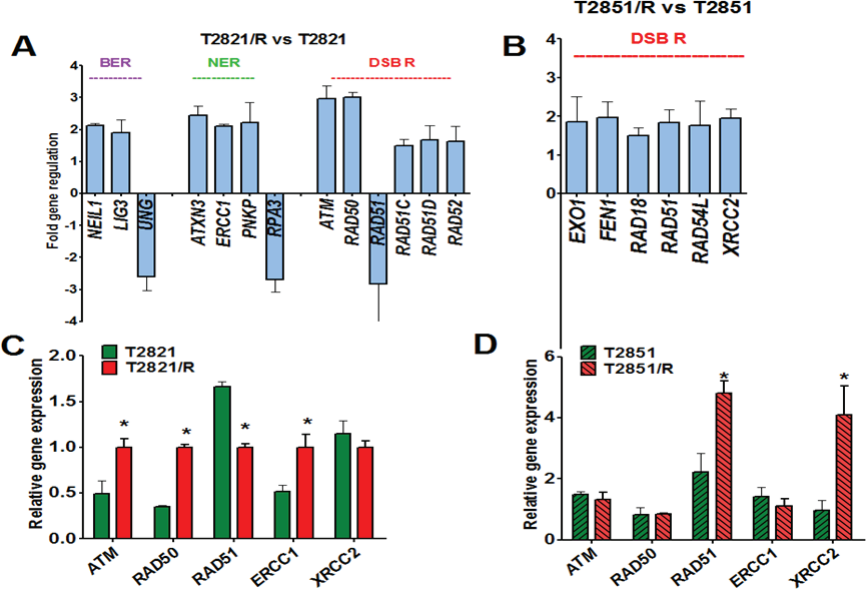
Changes in DNA repair genes associated with the IR-resistant phenotypes of T2821/R and T2851/R cells Parental T2821 and T2851 lung adenocarcinoma cells and radioresistant T2821/R and T2851/R cells exponentially growing under physiologically normal conditions were harvested. (**A, B**) The Human DNA Repair RT^2^ Profiler™ PCR Array kit (Qiagen), was used. Real-time PCR detection was carried out per the manufacturer's instructions. Genes involved in Base Excision Repair (BER), Nucleotide Excision Repair (NER), Double-Strand Break Repair (DSB R) which were found up/down regulated by more than 1.5 fold in T2821/R (A) and T2851/R (B) cells are presented. (**C, D**) Analysis of ATM, RAD50, RAD51, ERCC1 and XRCC2 genes expression in T2821 and T2821/R (C) and T2851 and T2851/R (D) cells; mRNA levels in each sample were determined by quantitative real-time PCR Comparative quantification of gene expression was performed using the ΔΔCT method. Expression of all genes is referenced to T2821B mRNA. *denotes significant differences between groups of tumor tissues at *p* < 0.05

To further analyze the differences between T2821/R and T2851/R cells and their respective parental T2821 and T2851 cells, expression of DNA repair genes was investigated (Figure 4). All cells were cultured under the physiologically normal adherent conditions. We used a Human DNA Repair PCR Array from SABiosciences to compare the expression of 84 key genes involved in base-excision repair (BER), nucleotide excision repair (NER), mismatch repair (MMR) and double -strand break repair (DSB R) processes. Altered gene profiles were detected in both T2821/R cells and T2851/R cells in comparison with the respective parental cells, with the majority of the altered genes being up regulated (Figure 4). Higher levels of gene upregulation were detected in T2821/R cells as compared with T2851/R cells. Detected gene changes in T2821/R cells included upregulation of 10 genes involved in BER, NER, and DSB R processes and downregulation of 3 genes including *UNG, PRA3* and *RAD51* (Figure 2A). In comparison, 6 genes were upregulated in T2851/R cells. Surprisingly, there was no overlap in gene changes between the T2821/R and T2851/R cells. The highest levels of gene upregulation were determined for the Ataxia Telangiectasia Mutated (*ATM)* and *RAD50* genes found inT2821/R cells. Levels *of ATM, RAD50, RAD51, ERCC1* and *XRCC2* genes expression that were identified by RT-PCR array were also validated by traditional QRT-PCR. *ATM, RAD50 and ERCC1* genes expression was significantly higher, but *RAD51* expression was significantly downregulated in T2821/R cells in comparison with parental T2821 cells *XRCC2* gene was expressed similarly in T2821 and T2821/R cells (Figure 4C). In contrast, T2851/R cells express higher levels of *RAD51 and XRCC2* genes, where as *ATM, RAD50 and ERCC1* genes were expressed similarly in T2851 and T2851/R cells (Figure 4D).

Taken together, these data indicate that multiple changes in the expression of DNA repair genes occurred in cells surviving fractionated IR treatment and these changes differed between the two cell lines.

### Differential response of T2821/R and T2851/R cells to IR

**Figure 5:**
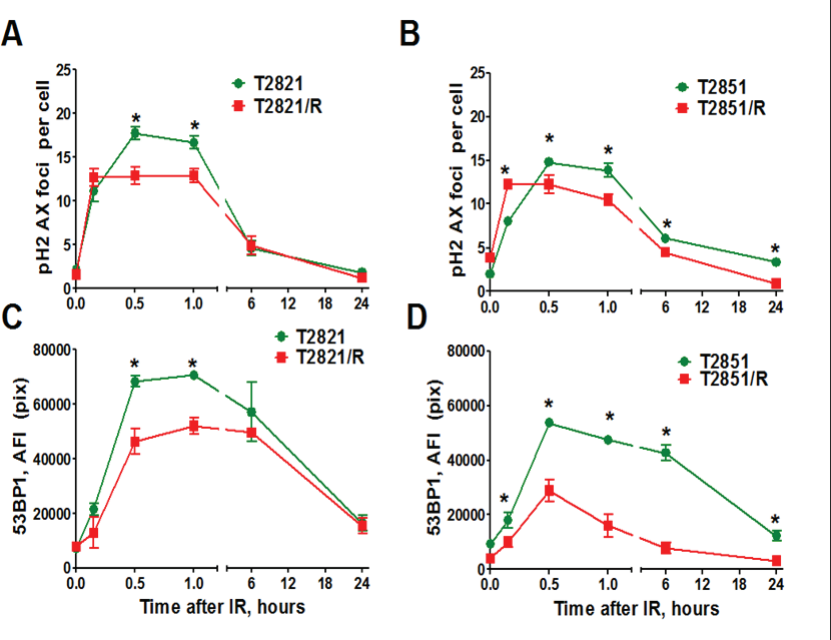
Analysis of γH2AX and 53BP1 foci Tumor cells were irradiated (5 Gy) and cultured for 0–24 h before fixation. Then the cells were immunofluorescently stained for γH2AX or 53BP1 and with Hoechst33342 and analyzed using HCA/HCS methods. (**A, B**) IR-induced γH2AX foci formation and foci duration in parental T2821, T2851 and IR-resistant T2821/R and T2851/R cells. Average numbers of the γH2AX foci per cell in cells treated with IR, 5 Gy, are shown at different time post -radiation. (**C, D**) The total average fluorescence intensities of 53BP1 in parental T2821, T2851 and IR-resistant T2821/R and T2851/R cells determined at 0–24 h post-radiation. Each point represents average data for 3000 cells. Results are expressed as the mean ± S.D. of three independent experiments.

To investigate, whether radioresistance in T2821/R and T2851/R cells (confirmed by clonogenic survival data, Figure 1B–1C) is due to higher DNA –DSB repair after irradiation, we used fluorescence staining and HCS/HCA methods as we described previously [28]. These techniques allow us to evaluate the dynamics of the γH2AX and 53BP1 foci as indicators of DSBs. The γH2AX foci were visualized as bright fluorescence spots and were found to be present in all IR-treated cells. Control T2821/R and T2851/R cells displayed similar levels of γH2AX foci as was seen in control T2821 and T2851 cells (Figure 5A, 5B). IR (5 Gy) induced γH2AX foci formation at 0.2–1 h post-radiation in all tumor cells, however the levels of γH2AX foci in T2821/R and T2851/R cells were significantly lower when compared to the parental T2821 and T2851 cells. Six hours post-radiation, the numbers of γH2AX foci were reduced by approximately 1/3 of the level detected at 1 h, suggesting that by this time most DNA repair had already occurred. The T2821/R cells and parental T2821 cells demonstrated similar levels of γH2AX foci at 6, and 24 hours post-radiation, while the T2851/R cells had lower levels of γH2AX foci as compared to parental T2851 cells (Figure 5A, 5B).

**Figure 6:**
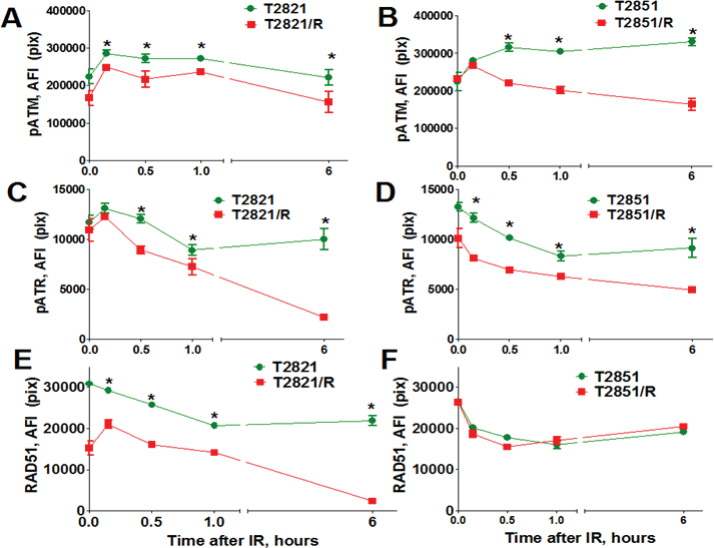
Analysis of pATM, pATR, and RAD51 expression Parental T2821 (**A, C, E**) and T2851 (**B, D, F**) cells and radioresistant T2821/R (**A, C, E**) and T2851/R (**B, D, F**) cells were irradiated (5 Gy) and cultured for 0–6 h before fixation. Then the cells were immunofluorescently stained for pATM (Ser1981) (A, B), or pATR (Ser473) (C, D), or RAD51 (E, F) and with Hoechst33342 and analyzed using HCA/HCS methods. The total average fluorescence intensities of proteins in parental and radioresistant cells are shown. Each point presents the average intensities (pixels) estimated for 3000 cells.

Next, 53BP1 foci were compared in parental and radioresistant cells at different time points post-irradiation. Both T2821/R and T2851/R cells showed lower level of 53BP1 expression in comparison with parental T2821 and T2851 cells (Figure 5C, 5D). However, T2851/R cells showed a dramatic reduction of 53BP1 after the first 30 min post-irradiation, suggesting that T2821/R and T2851/R cells have developed different radioresistance mechanisms. Therefore, next we investigated the status of a few key DNA repair proteins: pATM, pATR (ataxia telangiectasia and Rad3-related), and RAD51 in the parental and radioresistant AC cells after gamma-irradiation with dose of 5 Gy (Figure 6). IR treatments resulted in upregulated pATM expression in both parental and IR-resistant cells, however the total amount of phosphorylated protein was significantly lower in T2821/R and T2851/R cells than in parental T2821 and T2851 cells (Figure 6A, 6B). The levels of pATR were lower in both T2821/R and T2851/R cells as compared with the respective parental cells (Figure 6C, 6D).

RAD51 is a major protein involved in homologous recombination (HR) of DNA strand breaks. [44]. Interestingly, we found that cells growing in physiologically normal conditions have differing levels of expression for the RAD51 gene: while RAD51 was upregulated in T2851/R, RAD51 was downregulated in T2821/R cells in comparison with respective parental cells (Figure 4). Immunofluorescent detection of the RAD51 protein in gamma-radiated cells confirmed that RAD51 protein level in T2821/R cells was significantly lower than RAD51 protein level in parental T2821 cells (Figure 6E). The level of RAD51 expression in T2851/R cells and the post-radiation dynamics of expression were exactly the same as in parental T2851 cells (Figure 6F). These data provide more evidence that different radioresistance mechanisms were utilized in T2821/R and T2851/R cells.

Taken together, our data revealed that treatment of human lung adenocarcinoma cells with multiple fraction of IR leads to generation of residual radioresistant cells characterized by multiple changes in expression of genes/proteins involved in regulation of the cell cycle, EMT and DNA repair. These radioresistant cells were also found to be significantly more resistant to cisplatin; they have upregulated multiple factors that are involved in prosurvival signaling, such as AKT, inflammatory cytokine IL-6, growth and angiogenic factors, PDGFB, and SDF-1 (CXCL12), and CXCR4 receptor for SDF-1.

We hypothesize that multiple prosurvival proteins could be targeted in order to reduce the residual radioresistant cells which survive fractionated irradiation treatments.

### Effect of HSP90 inhibition on T2821/R and T2851/R cells

Cancer cell transformations are dependent on molecular chaperone HSP90, and inhibition of HSP90 leads to inappropriate processing of multiple client proteins involved in cell survival pathways [29, 34, 45]. AKT, which is significantly upregulated in both T2821/R and T2851/R cells, (Figure 1E) is among the client proteins for HSP90 [46]. We recently reported that IR treatment upregulated the level of pAKT in T2821 and T2851 lung adenocarcinoma cells, which could be blocked by the HSP90 inhibitor ganetespib [28].

**Figure 7:**
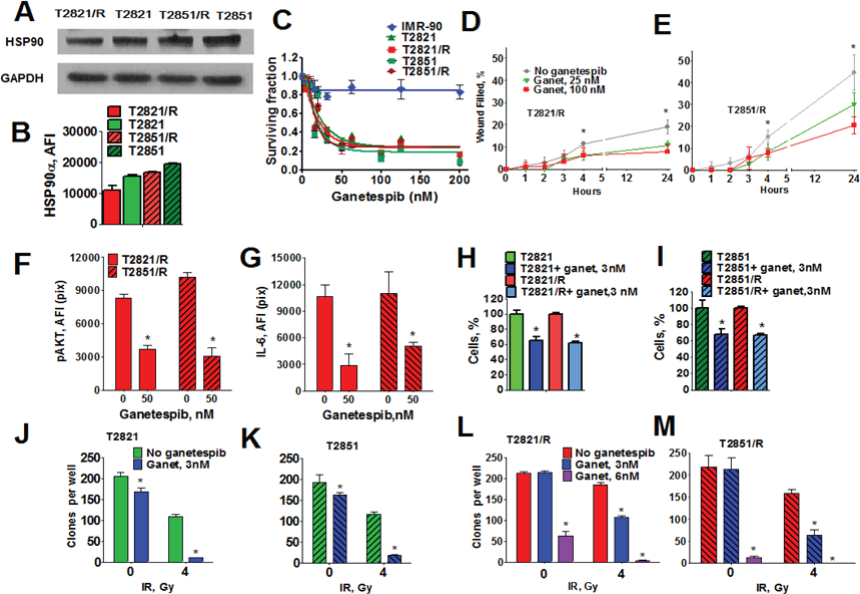
Effect of HSP90 inhibitor ganetespib on radioresistant cells (**A, B**) Analysis of HSP90 expression in parental and radioresistant lung AC cells. (A) Western blots analysis of HSP90 expression in radioresistant T2821/R and T2851/R cells and parental T2821 and T2851 cells. Cells were grown under physiologically normal conditions; cell lysates were prepared and immunoblotted using antibodies against HSP90a. GAPDH was included as a control. (B) Tumor cells were grown in 96 well plates, fixed, stained for HSP90 and also stained with Hoechst 33342. The average fluorescence intensities of HSP90 in parental T2821 and T2851 cells and radioresistant T2821/R and T2851/R cells are shown. Each point presents the average intensities (pixels) estimated for 3000 cells. (**C**) Ganetespib inhibits the proliferation of parental and radioresistant lung AC cells while not inhibiting normal human lung fibroblasts, IMR-90 cells. Cells were grown in the presence of ganetespib (0–200 nM) and the cellular viability was assessed after 72 h using a MTT assay. (**D, E**) Ganetespib reduces the motility of T2821/R and T2851/R cells. Migratory capacity was assessed by a wound healing assay. The migratory rates were determined by measuring wound width as a function of time. Data are expressed as the mean ± SD of three–six experiments (**F**) Ganetespib downregulates p-AKT expression in IR-resistant cells. Cells were treated with ganetespib (0 or 50 nM), for 48 hours, fixed and stained for p-AKT, and with Hoechst 33342. Cell images were acquired and analyzed by a Cellomics ArrayScan HCS Reader. The total average fluorescence intensities of p-AKT are shown. (**G**) Ganetespib reduces production of IL-6 in IR-resistant cells. Cells were grown in culture media supplemented with ganetespib (0, or 50 nM) and with monensim, 2 μM, for 48 hours. Cells were fixed, and immunofluorescently stained for IL-6 and with Hoechst 33342. Cell images were acquired and analyzed using the Cellomics ArrayScan HCS Reader (40X objective). The total average fluorescence intensities of IL-6 in cells are shown. The fluorescence intensities of respective IgG controls were subtracted. Each point presents average intensities (pixels) estimated for 3000 cells. (**H, I**) Ganetespib, 3 nM, inhibits proliferation of T2821 and T2821/R (H) and T2851 and T2851/R (I) cells. Cells were seeded in 96 well plates (3000cells/well) and grown for 72 h in presence of ganetespib (0, or 3 nM). Cells were fixed, stained with Hoechst 33342 and counted using the Cellomics ArrayScan HCS Reader (10X objective). Data are presented as % of control ± SE. (**G–M**) Ganetespib at low nanomolar concentrations sensitized T2821 (**J**), T2851 (**K**), T2821/R (**L**) and T2851/R (**M**) cells to ionizing radiation. Cells were pretreated with ganetespib (0, 3, 6 nM) for 24 h, irradiated (0, or 4 Gy) incubated for 7 days and then clonogenic survival was assessed.

Here we show that HSP90 protein is present at high levels in parental T2821 and T2851 cells as well as in radioresistant T2821/R and T2851/R cells (Figure 7A, 7B), suggesting that targeting HSP90 activity with ganetespib might reduce IR resistant cells. We further investigated whether ganetespib as a monotherapy would be an effective tool against IR-resistant lung adenocarcinoma cells and have evaluated the safety of ganetespib for normal diploid human lung fibroblasts (IMR-90 cells). T2821, T2851, T2821/R, T2851/R lung adenocarcinoma cells and normal human fibroblasts (IMR-90) cells were cultured in the presence of graded concentrations of ganetespib (0–200 nM) for 72 hours and the MTT assay was applied (Figure 7C). Ganetespib at concentration up to 200 nM did not affect viability of normal human fibroblasts. In contrast, ganetespib was found to have a strong antiproliferative effect against lung adenocarcinoma cells; in both the parental and IR-resistant cells (Figure 7B). Expression of EMT markers, upregulation of CXCR4 receptors and high migratory capacity is a key characteristic of metastatic tumor cells [47]. We have reported recently that ganetespib inhibits migration of T2821 and T2851 lung adenocarcinoma cells [28]. Here, using a scratch-wound assay, we demonstrate that ganetespib at concentrations of 25 nM and 100 nM inhibits the migration of T2821/R and T2851/R radioresistant cells (Figure 7D, 7E).

Treatment of T2821/R and T2851/R cells with ganetespib for 48 hours significantly downregulated p-AKT expression (Figure 7F) and decreased the level of IL-6 production (Figure 7G) in IR-resistant cells. Incubation of T2821 and T2851parental cells and radioresistant T2821/R and T2851/R cells with ganetespib at 3 nM concentration reduced cells proliferation (Figure 7H, 7I).

Next, using clonogenic survival assay we have determined that pretreatment of parental T2821 and T2851 cells and radioresistant T2821R and T2851/R cells with ganetespib at low concentration of 3 nM for 24 hours before irradiation significantly potentiated the effect of IR (Figure 7J–7M). Pretreatment of T2821/R and T2851/R cells with ganetespib at concentration of 6 nM reduced clonogenic survival of non irradiated cells and completely abrogated clonal survival after IR treatment with dose of 4 Gy (Figure 7L, 7M).

## DISCUSSION

Although significant advances in the use of high-dose IR have shown high efficacy in control of lung cancer tumors, patients were found to still suffer from distant metastasis [48, 49].

Chronic exposure to IR induced an adaptive response in the tumor cells resulting in tolerance to subsequent IR treatments with the generation of radioresistant residual cells that increase the risk of tumor recurrence, metastasis and negatively affects survival outcomes [31, 49, 50].

Therefore, it is of critical importance to eliminate these radioresistant residual cells.

To address this question we have generated T2821/R and T2851/R radioresistant sublines by treating T2821 and T2851 cells with multiple fractions of IR and have characterized their radioresistant phenotypes. Importantly, our study demonstrates the potent ability of the novel HSP90 inhibitor ganetespib to eliminate these radioresistant residual cells.

Currently the development of innovative pharmacotherapy for NSCLC is tailored to tumor-specific molecular aberrations, however radiotherapy treatments still are applied without stratification for the genetic background or genetic heterogeneity of the disease [48, 49, 51–53].

In our study we used 2 genetically dissimilar human lung adenocarcinoma cell lines: EGFR mutant (exon 21 L858R mutation) T2851 cells and T2821 cells expressing wild type (wt) KRAS and EGFR. To generate radioresistant sublines, T2821 and T2851 lung adenocarcinoma cells were both simultaneously treated with increasing doses of fractionated gamma-radiation. In this study we highlight not only similarities but also some of the diverse biological differences between T2821/R and T2851/R sublines which probably result from the differences in the genetic background of the tumor cells [28].

### Similarities between T2821/R and T2851/R cells

Both T2821/R and T2851/R cells have demonstrated higher clonogenic survival after irradiation in comparison with parental T2821 and T2851 cells. This observed increased clonogenic survival is associated with lower levels of γH2AX, 53BP1, pATM and pATR proteins in the resistant cells.

Importantly that both 2821/R and T2851/R cells surviving multiple fractions of IR display not only high resistance to ionizing radiation, but are also resistant to cisplatin. This is an essential observation since cisplatin and carboplatin are commonly used in combination with radiotherapy for NSCLC treatment [2–4].

Radioresistance and cisplatin resistance of T2821/R and T2851/R cells is associated with EMT phenotypes of these cells: cell morphology changes with the activation of EMT regulating transcription factors and expression of EMT associated proteins, vimentin and fibronectin. In cancers, EMT is associated with resistance to chemotherapeutic drugs and radiation [54, 55], via activation of the PI3K/Akt/mTOR pathway [56].

T2821/R and T2851/R cells have significantly upregulated AKT protein suggesting that targeting PI3K/AKT/mTOR signaling might radiosensitize cells and improve the outcome for radiotherapy. As a major regulator of the PI3K/Akt/mTOR pathway, Akt is a serine/threonine protein kinase that plays a critical role in suppressing apoptosis by regulating its downstream pathway. mTOR acts as a downstream effector for Akt, and regulates key processes such as cell growth and proliferation, cell cycle progression and protein translation through two distinct pathways: one involving the ribosomal p70S6 kinase (p70S6K), and the other involving eukaryotic translation initiation factor 4E (eIF4E)-binding proteins (4E-BPs) [57]. PI3K/AKT signaling is activated in response to IR treatment in NSCLC cells [28, 34]. Inhibition of PI3K/AKT/mTOR pathways has been evaluated as a potential strategy to sensitize tumor cells to radiation in different cancer cell lines, however the inhibitors that are currently available are effective in only relatively high concentrations [58].

Both T2821/R and T2851/R cells demonstrated an elevated production of interleukin IL-6, and platelet derived growth factor B (PDGFB). IL-6 is a pleotropic proinflammatory cytokine that has been shown to be expressed in over 50% of NSCLC and is associated with poor survival of lung cancer patients [59]. Studies of the IL-6 mechanism of action suggests that IL-6 promotes lung cancer cell proliferation and migration through activation of the transcription factor Signal Transducer and activator of Transcription 3 (STAT3) [35]. PDGFBB and its receptors play an important role in tumor cell migration and vasculature formation. These molecules are well known drivers of mesenchymal cell proliferation [60, 61] and are known clients for HSP90 [62].

A significant upregulation of SDF-1/CXCR4 was detected in both the T2821/R and T2851/R cells. These radioresistant cells were found to have elevated production of SDF-1 (CXCL12) and significant upregulation of CXCR4 receptors in comparison with their respective parental cells. Activation of SDF-1/CXCR4 signaling is a key characteristic of metastatic tumor cells [63, 64]. SDF-1/CXCR4 signaling is regulated by hypoxia inducible factor 1 (HIF1) [65, 66] which is also a client for HSP90 [17].

### Differences between T2851/R and T2851/R cells

While T2821/R cells have a growth rate similar to parental T2821 and T2851 cells, the doubling time for T2851R is significantly longer. Both T2821/R and T2851/R cells have a distinct spindle –like morphology and have lost cell-to cell junctions. These cellular morphological changes are associated with the epithelial-mesenchymal transition (EMT) but are a product of different patterns of gene expression in the two radioresistant cell lines. The acquired radioresistance in T2821/R and T2851 /R cells is associated with the multiple changes in genes and protein expression. While T2821/R cells show altered expression of genes related to BER, NER and DSB repair, T2851/R cells demonstrated changes only in DSB repair genes. Our data on DNA repair gene expression is consistent with previous studies of the global gene expression in parental and isolated radioresistant subpopulations in breast [67], esophageal [68], cervical [69], pancreatic [70] and lung cancers [71, 72] showing that acquired radioresistance is associated with upregulation/downregulation of hundreds of genes. These genes are involved in different cell functions and are located in different cellular compartments. Interestingly, *RAD51* gene was downregulated in T2821/R cells and RAD51 protein level was also lower in T2821/R cells, in comparison the T2851/R cells show the same level of *RAD51* gene and RAD51protein expression as T2851 cells.

Cell-cycle checkpoints surveillance serves to protect cells from DNA damaging stresses [73]. Both, parental T2821 and T2851 as well as IR resistant T2821/R and T2851/R cells were arrested in G2/M phase after irradiation, however only T2821/R cells showed an additional increase at G1 fraction at 24 and 30 hours post-radiation.

Taken together, our data demonstrates that treating NSCLC with the multiple fractions of IR selects for the radioresistant residual cells. These radioresistant cells survive radiotherapy via activation of multiple prosurvival signaling pathways and radioresistance mechanisms. Key components of these prosurvival signaling pathways include such genes as AKT, STAT3, HIF1, PDGFR, ATR/CHK1 etc., which are all clients for molecular chaperone HSP90 [20].

### Effects of ganetespib on T2821/R and T2851/R cells

Our study is the first to demonstrate that the HSP90 inhibitor ganetespib, used as a monotherapy, has a strong cytotoxic effect on radioresistant residual cells which have survived multiple fractions of gamma-radiation. We have determined that ganetespib as monotherapy has the similar antitumor effect on parental T2821 and T2851 lung adenocarcinoma cells and T2821/R and T2851/R radioresistant residual adenocarcinoma cells which survived multiple treatments of IR.

A key factor for the clinical success of a drug is the ability to selectively target only tumor cells and not normal tissue cells, therefore our finding that ganetespib doesn't affect proliferation of normal human lung fibroblasts is important.

High migration and invasion capacities are key characteristics of metastatic tumor cells, however, drugs capable of blocking metastasis are not available yet [63]. Our finding that ganetespib at nanomolar concentration decreases the migration of T2821/R and T2851/R radioresistant cells complements our recent observation that ganetespib was able to reduce the migration of A549, T2821 and T2851 lung adenocarcinoma cells [28] and highlight a ganetespib as a possible antimetastatic drug.

HSP90 inhibitors can both directly or indirectly modulate the expression of multiple client proteins. We found that AKT, PDGF, IL-6 and SDF-1/CXCR4 proteins are significantly upregulated in T2821/R and T2851/R cells. Therefore we chose to investigate the effect of ganetespib on two central transcription factors: HIF-1a and STAT-3 associated with these signaling pathways. It is known that the AKT/mTOR pathway drives the synthesis of HIF-1 alpha protein in irradiated tumors [74]. SDF-1/CXCR4 axis is essential for pericyte recruitment within the PDGF-BB-overexpressing tumors [75]. The HIF-1α/SDF-1/CXCR4 pathway governs the primary and metastatic tumor microenvironments and organ-specific metastases in different malignancies [76, 77]. Upregulation of the IL-6/STAT3 pathway is associated with lung cancer progressions [35]. Moreover, the presence of an autocrine signaling loop between HIF-1a and STAT-3 was demonstrated in pancreatic cancer cell lines, where hypoxia mediates an increase in IL-6 production through HIF-1a leading to activation of STAT-3 [78]. Lastly, both HIF-1a and STAT-3 are HSP90 client proteins. We found that inhibition of HSP90 with ganetespib resulted in downregulation of p-AKT and decreased production of IL-6 in T2821/R and T2851/R cells thus confirming the role of PI3/AKT and IL-6/STAT-3 signaling in radioresistance phenotype in T2821/R and T2851/R cells surviving multiple fractions of IR. Recently the central role of HSP90 in regulation of both HIF-1a and STAT-3 and a strong antiangiogenic effect of ganetespib were confirmed in colorectal cancer [79].

Of particular significance is our finding that ganetespib at 3 nM concentration has a strong radiosensitizing effect, while ganetespib at 6 nM concentration completely inhibits clonal growth from T2821/R and T2851/R cells radioresistant cells after IR, 5 Gy.

These data with our recent report [28] demonstrated that ganetespib effectively radiosensitizes A549, T2821 and T2851 lung adenocarcinoma cells *in vitro* and also reduced growth of T2821 tumor xenografts in mice and sensitized tumors to IR, altogether highlight the promise of combining ganetespib with IR therapies in the treatment of AC lung tumors.

## CONCLUSION

We established T2851/R and T2821/R radioresistant residual sublines by exposing genetically dissimilar human lung adenocarcinoma cell lines to multiple fractions of IR. These radioresistant cell sublines exhibited morphological changes and upregulation of multiple radioresistance mechanisms. Importantly that both 2821/R and T2851/R cells surviving multiple fractions of IR display not only high resistance to ionizing radiation, but are also resistant to cisplatin. The radioresistance mechanisms detected include alterations of cell cycle distribution, upregulation of PI3K/AKT signaling, higher expression of DNA repair genes, and elevated production of prosurvival and angiogenic factors (IL-6, PDGFB and SDF-1) and CXCR4 receptors important for lung cancer progression and metastasis.

We have presented data which demonstrates that the inhibition of HSP90 is a unique approach which is able to sensitize lung tumor cells to fractionated IR and avoid selection of residual radioresistant cells. Avoiding selection for residual radioresistant cells is significant as we believe these cells might be responsible for tumor recurrence and metastasis.

## MATERIALS AND METHODS

### Cells

We used T2821 and T2851 lung AC cells recently established from the surgical tumor samples [26]. T2821 and T2851 AC cells were cultured in RPMI media that was supplemented with 10% FBS. Human diploid fibroblast cell line IMR-90 from American Type Culture Collection (ATCC CCL-186, USA) was cultured in DMEM culture media that was supplemented with 10% FBS.

### Reagents

Hoechst 33342 and anti-β-actin and anti-SDF-1 antibodies were purchased from Sigma-Aldrich (Sigma-Aldrich, St. Louis, MO). Draq5^TM^ was purchased from eBioscience (San Diego, CA). Antibodies against ATR, RAD51, IL-6R, GP130, pATM (Ser1981), TWIST1, SNAIL1, N-cadherin and vimentin were from Abcam Inc. (Abcam, Cambridge, MA). Antibody against fibronectin was obtained from BD Pharmigen (BD Biosciences, San Diego, CA). ATM, AKT, pAKT (Ser 473), pATR (Ser428), ZEB1, SNAIL2 (SLUG), E-cadherin and β-catenin antibodies were purchased from Cell Signaling Technology Inc., (Danvers, MA). Antibodies against 53BP1 and γH2AX (Ser139) were obtained from EMD Millipore (Darmstadt, Germany). Rad51, PDGFB and ATR antibodies were from Santa Cruz biotechnology, (Dallas, TX). CXCR4, PDGFRα and PDGFRβ antibodies and human IL-6 ELIZA kit were from R&D (Minneapolis, MN). Secondary antibodies conjugated with Alexa^®^ −488, −546, and −680 were from Molecular Probes (Invitrogen, Carlsbad, CA).

HSP90 inhibitor ganetespib [3–(2,4–dihydroxy–5–isopropylphenyl)–4–(1–methyl–1H–indol–5–yl)–1H– 1,2,4–triazol–5(4H)–one] was generously provided by Synta Pharmaceuticals Corp. (Lexington, MA).

### Irradiation

AC cells were irradiated, as cell suspension or as monolayer, using the Shepherd Mark 168 Irradiator, (^137^Cs Irradiator) (JL Shepherd, San Fernando, CA, USA) at a dose rate of 70.6 rad/min at room temperature.

### Cell's doubling time

Cells were plated into 24 well plates and cell numbers per well were estimated at 8, 24, 48 and 72 hours. Cell numbers per well were summarized by mean ± standard deviation for each of the 4 cell groups (i.e., T2821, T2821 /R, T2851 and T2851/R) at each time of measurement. A simple linear regression model was built for log (cell number per well) for each cell group, using the measurement time as a continuous explanatory variable. For each cell group, the estimate of cell doubling time (dt) was calculated based on the slope of regression line (β) using the formula dt = log2/β, and its standard error was also calculated, based on the formula: SE(dt) = log(2)*SE(β)/β2 [80]. The comparison of doubling times between cells groups was performed using *z*-tests, based on these estimated values and their standard errors.

### Cells staining procedure for cellomics array scan automated imaging

Cells were fluorescently stained as described [31]. Cell images were acquired using the Cellomics ArrayScan HCS Reader (Cellomics/ThermoFisher, Pittsburgh, PA) and were analyzed using the Compartment or Target Activation BioApplication Software Modules. The Cellomics ArrayScan HCS Reader was utilized to collect information on the distribution of fluorescently labeled components in stained cells. Data was captured, extracted and analyzed with ArrayScan II Data Acquisition and Data Viewer version 3.0 (Cellomics),

### Cell proliferation and viability assays

Cells (5000 cells/well) were seeded in triplicate in 96 well−plates and incubated for 72 h in the absence or presence of different concentrations of ganetespib (0–200 nM) or cisplatin (0–20 μM). The number of viable cells was determined by using MTT conversion assay, according to the manufacture's protocol (Sigma-Aldrich). In some experiments, cells were counted using the Cellomics ArrayScan HCS Reader (10x objective) after staining with 2 μg/ml of DNA binding dye, Hoechst 33342, for 20 min.

### Analysis of the intracellular cytokines in tumor cells

To detect intracellular cytokines production and accumulation, we used the methods as previously described [39]. Parental T2821, T2851 cells and radioresistant T2821/R and T2851/R cells (10^4^cells/well) grown in 96-well plates were incubated with monensin (2 μM) for 48 hours. Cells were fixed in 2% PFA, washed, permeabilized with 0.1% Triton X-100, washed with FACS buffer and then incubated with antibodies against IL-6, SDF-1 (CXCL12) and PDGFB for 1 hr and with secondary antibodies conjugated with Alexa 488, or 546 fluorochromes (Molecular Probes/Invitrogen, Eugene, CA) for 1 hr. Cell nuclei were stained with Hoechst 33342 at 2 μg/ml for 20 min to identify individual cells. Cell images were acquired and analyzed using the Cellomics ArrayScan HCS Reader (Cellomics/ThermoFisher, Pittsburgh, PA).

### Measurement of IL-6 secreted by tumor cells

T2821, T2851, T2821/R and T2851/R cells were seeded in 96-well plates (3×10^3^cells/well) and grown for 72 hours. Each cell line was plated in three independent wells. Then culture media conditioned by tumor cells were collected, cells were fixed, stained with Hoechst 33342 and cell numbers in each well were counted using Cellomics Array.

ELISA kit was obtained from R & D Systems (Minneapolis, MN, USA) and concentrations of IL-6 were determined according to manufacturer's instructions. The IL-6 concentrations were calculated as pg/1 × 10^6^ tumor cells as follows:

$$\frac{\text{Concentration of IL-6 (pg/ml)}\times \text{volume of media (ml/well)}\times \text{1}\times {\text{10}}^{6}\text{ cells}}{\text{Number of tumor cells per well}}$$

### Cell cycle analysis

Exponentially growing cells were irradiated (0, or 5 Gy) and fixed after 0, 8, 24 and 30 hours post-radiation. Cells were then harvested and resuspended in ice cold PBS. Iced cold 70% ethanol was used to fix the cells and they were stored overnight at 4°C, washed twice with PBS, and resuspended in 50 μg/ml PI staining reagent containing 100 μg/ml RNase and 0.1% Triton X-100 for 30 min in the dark. Cells were analyzed by flow cytometry (Accury C6, Becton-Dickinson) and the percentage of cells in the different phases of the cell cycle was analyzed with Becton-Dickinson software.

### *In vitro* clonogenic assays

Exponentially growing cells were harvested by exposure to trypsin and cells were seeded in 6 well plates, 500 cells per well. Next day, HSP90 inhibitor ganetespib was added to the cultures at the final concentrations of 0–10 nM. 24 hours later, the plates were irradiated using a 137Cs gamma-ray source with doses ranging from 0 to 10 Gy. Seven days later, the cells were fixed and stained with crystal violet; colonies ≥ 50 cells were counted and the size of the colonies were measured (Gel Count colony counter, Oxford Optronix, Oxford, UK) as we described [31]. Data was analyzed with linear quadratic and single-hit multi-target models [81].

### Immunofluorescence detection of γH2AX, pATR, pATM, RAD51, and 53BP1

Cells were irradiated with 0 or 5 Gy at RT. After IR treatment, cells were incubated in the culture media supplemented with 10% of FBS, 37^ο^C, 5% CO_2_, for 0–24 hours. Then the cells were fixed and permeabilizedATM, phospho-ATM and RAD51 antibodies and secondary antibodies conjugated with Alexa 488, and 546. HCS/and HCA ArrayScan tools were used as described previously [26].

### Real-Time PCR and gene expression profiling

RNA was isolated from the cells using the RNeasy kit from Qiagen (Germantown, MD), and the extracted RNA was converted to cDNA using the qScript™ cDNA SuperMix from Quanta Biosciences (Gaithersburg, MD) according to manufacturer's protocols. Real-time PCR was carried out using the Fast SYBR^®^ Green Master Mix (Life Technologies) and was performed on the StepOnePlus™ Real-Time PCR System (AB Applied Biosystems, Foster City, CA). Primers for human *SNAIL1, SNAIL2*, TWIST1, ZEB1, ZEB2, CDH1 (E-cadherin), CDH2 (N-cadherin), ATM, RAD50, RAD51, ERCC, and XRCC2 genes were purchased from IDT Technologies (Coralville, IA), Amplifications were run in triplicate for each primer pair. Comparative quantification of gene expression was performed using the ΔΔCT method. Expression of all genes is referenced to T2821 mRNA. List of primers sequences is presented in Supplemental Table 1.

The Human DNA Repair RT^2^ Profiler™ PCR Array kit (Qiagen, Germantown, MD), was used. This PCR Array profiles the expression of 84 key genes encoding the enzymes that repair damaged DNA. This array represents genes involved in the base-excision, nucleotide excision, mismatch, double-strand break, and other repair processes. Real-time PCR detection was carried out per the manufacturer's instructions. The experimental cocktail was prepared by mixing cDNA isolated from cell lines with the RT2 Real-Time SyBR Green/ROX qPCRMasterMix. The mixtures were equally aliquoted into the 96-well plate containing predispensed gene-specific primer sets, then real-time PCR was performed using the StepOnePlus™ Real-Time PCR System (ABI, Applied Biosytems Corp., Foster City, CA). Differences in genes expression were compared between T2821 and T2821IR/R cells, as well as T2851 and T2821IR/R cells. Analyses of the raw data were done through the GeneGlobe Data Analysis Center Web (Qiagen, Germantown, MD). Experiments were performed three times.

### Western blotting

Western blotting analysis was performed as previously described [28]. The tumor cells were harvested and disrupted using lysis buffer (Cell Signaling Technology) in the presence of protease inhibitor and incubated on ice for 30 minutes (vortex every 10 min). Lysates were clarified by centrifugation and equal amounts of proteins resolved by SDS-PAGE before transfer to PVDF membrane (Bio Rad, Hercules, CA). The membranes were blocked with 5% non-fat milk, incubated with primary antibodies against human AKT or HSP90 followed by addition of secondary antibodies. Chemiluminescence signals were detected on the X-ray film. As an internal control the GAPDH primary antibody was also probed. The gel-digiting software Un-Scan-It gel (Version 5.1; Silk Scientific, Inc., Orem, UT, USA) was used to quantify the intensities of Chemiluminescent bands detected.

### Statistical Analysis

Data are presented as mean ± SD. Comparisons between values were performed using a two tailed Student's t-test. For the comparison of multiple groups, a one- or two-way ANOVA test was applied. For all statistical analyses, the level of significance was set at a probability of P < 0.05. All experiments were repeated 3–5 times. IR survival curves were analyzed by comparison with the linear-quadratic and the single-hit multi-target models. Statistical comp_a_risons used the final slope of the survivorship curves, representing multiple-event killing (D0) [82].

## SUPPLEMENTARY MATERIAL TABLE


