# Supplementary material for: Radioresistant human lung adenocarcinoma cells that survived multiple fractions of ionizing radiation are sensitive to HSP90 inhibition
Source: Oncotarget. 2015 Oct 27;6(42):44306–22. doi: 10.18632/oncotarget.6248 (PMC4792558; doi:10.18632/oncotarget.6248)
Supplement: Supplementary file 1 [file oncotarget-06-44306-s001.pdf]

Radioresistant human lung adenocarcinoma cells that survived multiple fractions of ionizing radiation are sensitive to HSP90 inhibition

Supplementary Materials

Supplementary Table 1: List of primers

	Gene name	Primer Sequence
1	*RAD51-F*	GGGAATTAGTGAAGCCAAAGC
	RAD51-R	TATGATCTCTGACCGCCTTTG
2	*ATM-F*	ATTCCGACTTTGTTCCCTCTG
	ATM-R	CATCTTGGTCCCCATTCTAGC
3	*RAD50-F*	CTGTTTGATGTTTGTGGTAGCC
	RAD50-R	TGGTTTTCGTCTGTTAGCTGAG
4	*ERCC1-F*	AATTTGTGATACCCCTCGACG
	ERCC1-R	TGTGAGATGGCATATTCGGC
5	*XRCC2-F*	CAGTTGGTGAATGGCGTTG
	XRCC2-R	CTACCTTCAAGTCGGGCAAG
